# Investigating hematuria among young Indian adults using MDCT urography

**DOI:** 10.6026/973206300200794

**Published:** 2024-07-31

**Authors:** Mohammed Musheer Ahmed, Holebasu B, Yogesh S, Prashanth A, Naveen Kumar Nallathambi, Arun K, Gaurav Mittal

**Affiliations:** 1 Department of Urology, Queen Alexandra Hospital NHS, United Kingdom; 2 Department of Radiology, KLE JGMM Medical College, Hubballi, India; 3 Institute of Internal Medicine, Madras Medical College and Rajiv Gandhi Government General Hospital, Chennai, India; 4 Department of Physiology, Mahatma Gandhi Institute of Medical Sciences, Sevagram, Wardha India; 5 Institute of Internal Medicine, Madras Medical College, Chennai, India; 6 Department of General Medicine, KarpagaVinayaga Institute of Medical Sciences & Research Centre, Tamilnadu, India; 7 Mahatma Gandhi Institute of Medical Sciences, Sevagram, Wardha, Maharashtra, India

**Keywords:** Urography, Hematuria, MDCT, Clinical Examination, Urolithiasis, Diagnostic Imaging

## Abstract

Hematuria may suggest bladder cancer, renal cell carcinoma, UUT-UCC, or urinary tract stones. Therefore, it is of interest to use Multi-Detector Computed Tomography (MDCT) to determine the cause of hematuria in children and connect MDCT results with cystoscopic and histological findings. The study included 110 young people under 40 with microscopic or macroscopic hematuria. A clinical exam and complete history were recorded. MDCT data from non-contrast and contrast-enhanced scans were properly documented. Histopathological and cystoscopic findings were noted alongside MDCT data when appropriate. The study's typical patient was 26 years old, 66% male. Eighty of the cases had hematuria as the cause. In 66 of 80 individuals, renal or ureteric calculi were the most common clinically relevant outcomes. There were four renal and four UB masses. Two cases of renal papillary necrosis and four of pyelonephritis/renal abscess were identified. MDCT diagnosed renal and vesical masses 100% accurately. MDCT can diagnose and treat hematuria, especially in young men, according to one study. The findings emphasize clinical awareness and targeted diagnosis. Further research is needed to determine hematuria causes and prevention across demographics.

## Background:

Young adults frequently have hematuria [1, 2- 3]. It is a fairly common symptom of urinary tract disease and can arise from any place in the urinary system. Hematuria can be tiny or gross. While the definition of microscopic hematuria is highly debatable and depends on a number of factors, such as the urine collection method, hematuria detection method, number of positive results, and patient characteristics, gross hematuria is concerning and has a high predictive value for malignancy [4]. The most frequent cause of painless hematuria in both young adults and the elderly is renal tumours and the urinary bladder (UB). An accurate history and physical examination are part of the evaluation process for a patient with hematuria [1]. Upper tract imaging, cystoscopy, and cytology are all part of a comprehensive assessment. There are several imaging modalities available, the most popular being computed tomography (CT) and ultrasonography (USG). When evaluating the kidneys and UB, non-invasive ultrasound (USG) is a safe and relatively reliable method [3]. On the other hand, it is not very good at identifying calculi and tiny renal and UB masses. For a single, thorough non-invasive assessment of the urinary tract, including UB, computed tomography (CT) is the preferred modality for assessing either microscopic or gross hematuria. Subsequent multi-detector computed tomography (MDCT) urography enables the identification of small renal and UB masses that may not be noticeable on upper extremity geodesy [5]. Therefore, it is of interest to use MDCT to identify the aetiology of hematuria in young people and to correlate the results with cystoscopy and histological assessment.

## Methodology:

## Study design and setting:

This prospective study was carried out in a north Indian tertiary care facility. The study was approved by the institution and the ethics committee before it started.

## Selection criteria:

The research comprised 110 young individuals (40 years of age or younger) who were instructed to undergo an MDCT of the abdomen and pelvis after presenting with gross or microscopic hematuria. Patients with renal and cardiovascular diseases, as well as those who declined to participate, were not included in the research.

## Data sources and variables:

Siemens Healthcare's Sonoma Definition AS+ 64-slice MDCT scanners were used to complete 100 MDCT exams in total. A three-scan CT routine, comprising an unenhanced scan, a nephrographic phase scan, and an excretory phase scan of the abdomen and pelvis following contrast injection, was used to scan patients while they were supine. In certain individuals, further delayed images were acquired based on observations made during the nephrographic and excretory phases. Three highly experienced radiologists examined the images. The results of the tests were noted, and the cause of the hematuria that was clinically significant was identified. A histological study was carried out on individuals who had both renal mass and UB. Results from MDCT were associated with histopathological results.

## Statistical analysis:

Statistical analysis was conducted using the data entered into a Microsoft Excel sheet, with results presented in both counts and percentages. This approach allowed for thorough examination of the dataset, enabling the identification of trends, patterns, and associations pertinent to the study objectives.

## Results:

Table 1 describes the distribution of cases across different age groups which reveals that the highest frequency is in the 21-30 age group, accounting for 42.73% of the total cases (n=110), with 32 males and 15 females. This is followed by the 31-40 age group, comprising 29.09% of the cases, with 20 males and 12 females. The 11-20 age group represents 22.73% of the cases, with 17 males and 8 females. The 0-10 age group has the lowest frequency, with 5.45% of the cases, including 5 males and 1 female. Overall, there are 74 males and 36 females, with a mean age of 25.02 years and a standard deviation of 8.64 years. Table 2 outlines the distribution of hematuria causes identified by MDCT in 80 cases which reveals that the majority of cases fall under the category of significant and requiring treatment. Specifically, renal and/or ureteral calculus accounts for 66 cases, pyelonephritis and/or renal abscess for 4 cases, and renal papillary necrosis for 2 cases. In the life-threatening category, there are 4 cases each of renal mass and vesical mass with a total of 8 cases.

## Discussion:

In a clinical environment, the initial indication of hematuria is often the presence of blood in the urine. It can be solitary or present in association with other urine abnormalities, asymptomatic or symptomatic, short- or long-term, and it can happen as a single finding or as a component of a broader pattern [5]. Haematuria is a frequent urinary tract pathology that should be addressed by the treating physician as well as the patient. Haematuria is an often-occurring sign of several urinary tract illnesses, such as renal parenchymal diseases, calculi, neoplasms, infections, trauma, medication-induced thrombocytopenia, developmental abnormalities, and infections [6]. The analysis of 110 cases of hematuria across different age groups, indicates a significant concentration of cases in young adults, particularly those aged 21-30 years, who account for 42.73% of the total cases. This group is followed by the 31-40 age group at 29.09% and the 11-20 age group at 22.73%. The lowest frequency is observed in the 0-10 age group, representing only 5.45% of the cases. The male predominance in these cases is evident, with 74 males compared to 36 females. The mean age of the patients is 25.02 years, with a standard deviation of 8.64 years, highlighting a relatively young cohort with hematuria. The American Urologic Association established practice guidelines in 2001 for the examination of individuals with hematuria. These guidelines advocate cystoscopy of the UB, urine cytology, and first upper urinary tract imaging using either excretory urography or CT urography [7, 8]. Compared to excretory urography, MDCT offers superior diagnostic accuracy for identifying urinary calculi, renal and UB masses, renal and perirenal infections, and unexplained extra-urinary illnesses [9, 10, 11-12]. Additionally, the American College of Radiology has advised against using excretory urography for evaluating hematuria and instead recommends CT urography [12].

Eighty individuals in the current investigation had a clinically relevant cause of hematuria. There was a maximum of 66 instances of renal or ureteral calculi in this group. In addition, our study included 4 cases of renal mass and/or UB mass, 4 cases of pyelonephritis, and 4 cases of renal abscess. Urinary tract calculi, renal and vesical masses, and upper urinary tract infections comprised the majority of the study's clinically important results, which supports CT's superiority over other modalities [13]. The results of this investigation are consistent with those of investigations conducted by Ghous MH *et al.* [15] and Mahajan M *et al.* [14]. Renal or ureteral calculi were the most frequent cause of hematuria in both investigations. All instances of vesical and renal masses were diagnosed in the current study by MDCT, and the results were linked with pathological examination. Therefore, in the current investigation, the diagnostic accuracy of MDCT in assessing renal and vesical masses was 100%. Numerous studies have shown potentially fatal diseases on urinary tract imaging, which is why it is recommended that young individuals should have upper urinary tract imaging done when they have microscopic hematuria [16, 17, 18-19]. The small sample size of the current study-only 110 patients were included-was one of its shortcomings.

The radiation exposure is one of the main issues with using MDCT. Approximately 1.5 times the radiation risk from an intravenous urogram (IVU) is associated with the mean effective dose of CT urography, which is 14.8 mSv [20]. Because of the much greater radiation dose in young adults, as low as reasonably attainable (ALARA) guidelines dictate the prudent and careful use of MDCT in the examination of hematuria in young people. The demographic distribution and gender disparity observed in this study could be attributed to various etiological factors, including lifestyle, genetic predispositions, and environmental exposures. The predominance of males in the study may reflect a higher incidence of conditions like renal calculi among men, which warrants further investigation into gender-specific risk factors. The findings underscore the importance of MDCT in accurately identifying the causes of hematuria, particularly in young adults. The high prevalence of renal and ureteral calculi necessitates prompt diagnosis and management to prevent complications. Furthermore, the identification of life-threatening conditions such as renal and vesical masses, although less frequent, highlights the critical role of imaging in the early detection and treatment of potentially fatal pathologies.

## Conclusion:

MDCT can accurately diagnose and treat both common and severe hematuria problems. The bulk of hematuria cases in this cohort are young males, indicating the need for increased clinical awareness and targeted diagnostic techniques. Further research is needed to determine hematuria causes and prevention in different demographic groups.

## Figures and Tables

**Table 1:** Distribution of Age Groups, Gender and Percentage of Cases with Mean and Standard Deviation (n=110)

**Age Group**	**Frequency of Cases**	**Percentage (%)**	**Males**	**Females**
0-10	6	5.45%	5	1
Nov-20	25	22.73%	17	8
21-30	47	42.73%	32	15
31-40	32	29.09%	20	12
Total	110	100%	74	36
Mean: 25.02				
Standard Deviation: 8.64				

**Table 2:** Hematuria Causes Identified by MDCT (n=80)

**Category**	**Frequency of Cases**
Significant and Requiring Treatment	
A)Renal and/or ureteral calculus	66
B)Pyelonephritis and/or Renal Abscess	4
C)Renal Papillary Necrosis	2
Life Threatening	
A)Renal Mass	4
B)Vesical Mass	4
Total	80
